# Multiple infected ulcerative plaques in an alcohol addicted patient

**DOI:** 10.11604/pamj.2019.33.201.15389

**Published:** 2019-07-15

**Authors:** Felipe Tavares Rodrigues, José Augusto da Costa Nery

**Affiliations:** 1 Escola de Medicina e Cirurgia do Rio de Janeiro, Universidade Federal, Estado do Rio de Janeiro, Unirio, Brazil; 2 Instituto Oswaldo Cruz, Fundação Oswaldo Cruz, Fiocruz and Sanitary Dermatology Department of Santa Casa de Misericórdia do Rio de Janeiro, Unirio, Brazil

**Keywords:** Ulcerative plaques, alcohol addicted, purulent wounds

## Image in medicine

A 47-year-old male patient came to the Souza Araújo outpatient clinic of the Oswaldo Cruz Foundation, a reference center for treating leprosy, to confirm mycobacteriosis after living in close contact with a leprosy patient. The patient was emaciated, having lost 12 kg in 2 months, febrile and presented nystagmus. The patient had multiple erythematous-livedoid, hypoesthetic, ulcerated, and purulent wounds in light-exposed areas, which had appeared suddenly 2 months before admission and did not regress after amoxicillin therapy. The patient reported loss of sensitivity, paresthesia, and asthenia in the lower limbs. Adenomegaly and visceromegaly were not observed. The patient had an approximately 25-year history of alcoholism and smoking. The patient also reported having intermittent diarrhea. The patient was instructed to take 300 mg of niacinamide per day, ingest other B-complex vitamins, use amoxicillin-clavulanic acid to treat impetigo, reduce alcohol intake and improve nutrition. On the return visit one month later, the lesions and peripheral neuropathy had regressed significantly, although the patient had used B-complex formulations containing only 50 mg of niacinamide for financial reasons. Pellagra was described in eighteenth-century Europe by Gaspar Casal, related to poverty and low ingestion of animal products. It was a public health problem in the United States at the beginning of the last century, but pellagra is rare today and diagnosed particularly among individuals with alcohol abuse, taking some medications, with desorption syndromes and HIV. The classic triad of symptoms includes dermatitis, diarrhea and dementia. Scaly dermatitis is more common in areas exposed to sunlight.

**Figure 1:**
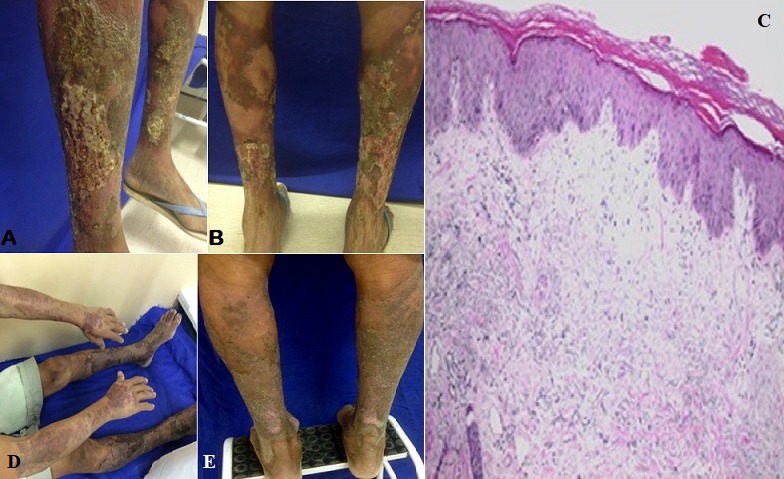
(A, B) multiple infected ulcerative and scaly lesions simetric distributed in both legs; C) histologic image showed parakeratotic hyperkeratosis, signs of epidermal hyperproliferation with ballooning and irregular acanthosis. We could see papilary dermal edema, inflammatory mononuclear cell infiltrate areas, extravasation of red blood cells and actinic elastosis. HE 10x Magnification; D) the previous dorsal legs lesions with healing crust aspect after treatment; E) widespread cicatricial spots distributed among light exposed body areas

